# Socioeconomic status of patients in a Swedish national self-management program for osteoarthritis compared with the general population—a descriptive observational study

**DOI:** 10.1186/s12891-019-3016-z

**Published:** 2020-01-06

**Authors:** Kristin Gustafsson, Joanna Kvist, Marit Eriksson, Leif E. Dahlberg, Ola Rolfson

**Affiliations:** 1 Unit of Physiotherapy, Department of Health, Medicine and Caring Sciences, Linköping University, Linköping, Sweden; 2 Department of Physiotherapy, Rehabilitation Centre, Ryhov County Hospital, Jönköping, Sweden; 3 Division of Physiotherapy, Department of Neurobiology, Care Sciences and Society, Karolinska Institutet, Stockholm, Sweden; 4 Department of Medical and Health Sciences, Linköping University and Futurum - Academy for Health and Care, Region Jönköping County, Jönköping, Sweden; 5 Department of Clinical Sciences Lund, Orthopedics, Faculty of Medicine, Lund University, Lund, Sweden; 6 Department of Orthopaedics, Institute of Clinical Sciences, Sahlgrenska Academy, University of Gothenburg, Gothenburg, Sweden

**Keywords:** Hip, Knee, Osteoarthritis, Self-management, Socioeconomic status, Registries

## Abstract

**Background:**

First-line treatment for hip and knee osteoarthritis (OA) including education and supervised exercises, delivered as a self-management program, is considered one of the mainstays in OA treatment. However, the socioeconomic profile of the population that utilizes first-line treatment for hip and knee OA is unclear. The aim of this study was to describe the socioeconomic status (SES) of a population referred to a self-management program for OA, in comparison with that of the general Swedish population.

**Methods:**

This is a cross-sectional study including 72,069 patients with hip or knee OA enrolled in the National Quality Register for Better management of patients with Osteoarthritis (BOA) between 2008 and 2016, and registered before participation in a structured OA self-management program. A reference cohort (*n* = 216,207) was selected from the general Swedish population by one-to-three matching by year of birth, sex and residence. Residential municipality, country of birth, marital status, family type, educational level, employment, occupation, disposable income and sick leave were analyzed.

**Results:**

The BOA population had higher educational level than the reference group, both regarding patients with hip OA (77.5% vs 70% with ≥10 years of education), and with knee OA (77% vs 72% with ≥10 years of education). Their average disposable income was higher (median [IQR] in Euro (€), for hip €17,442 [10,478] vs €15,998 [10,659], for knee €17,794 [10,574] vs €16,578 [11,221]). Of those who worked, 46% of patients with hip OA and 45% of the reference group had a blue-collar occupation. The corresponding numbers for knee OA were 51 and 44% respectively. Sick leave was higher among those with hip and knee OA (26%) than those in the reference groups (13% vs 12%).

**Conclusions:**

The consistently higher SES in the BOA population compared with the general population indicates that this self-management program for OA may not reach the more socioeconomically disadvantaged groups, who are often those with a higher disease burden.

## Background

Osteoarthritis (OA) of the hip and knee is among the leading causes of global disability [1, 2]. The prevalence of the disease is projected to increase rapidly because of an ageing and increasingly obese population [3], which will lead to greater demands on both healthcare services and the labor market. Strategies to prevent OA and reduce the burden of the disease will therefore become increasingly important [1].

According to guidelines, evidence-based first-line treatment of OA includes education, exercises and weight control [4, 5]. These treatments have been shown to reduce the impairments and disabilities caused by OA [4–7] and potentially to delay joint replacement surgery [6, 8, 9]. To overcome discrepancy between guidelines for OA treatment and practice, “Better Management of Patients with Osteoarthritis” (BOA), was initiated in Sweden in 2008. BOA includes three parts; 1) training of physiotherapist to deliver OA treatment according to guidelines, 2) first-line treatment of patients with OA, as a structured self-management program with theoretical group sessions and individually adapted exercises and 3) evaluation of patients included in the program in the National Quality Register BOA [5]. Health care, regardless of level of care, is in Sweden primarily financed through public taxes, with a maximum payment of approximately €109 for outpatient visits during a twelve month period, aiming to minimize the financed barriers for seeking health care [10].

Currently, it is difficult to predict deterioration in OA in order to identify individuals who will have slow disease development or will be in need of further interventions [11–13]. Still, to improve our understanding of the patients’ perception of health and disability, their utilization of care and their response to treatment, additional factors need to be considered [14]. Socioeconomic status (SES) is a multidimensional concept, reflecting both economic and social factors that influence the position held by individuals or groups within the structure of a society [15]. Previous research has revealed associations between SES and health for common chronic diseases, where the more socioeconomically disadvantaged individuals display poorer health [16]. Accordingly, the prevalence of OA is higher in individuals with low SES than in those with higher SES [17–19], and lower SES is associated with a higher disease burden for both hip and knee OA [14, 20–22]. An individual’s SES has also been shown to influence their access to general healthcare services and healthcare decision-making [10, 23]: individuals with OA and low SES, have poorer access to nonsurgical healthcare services such as self-management education, physical therapy and medication, and to joint replacement surgery [24, 25]. Low SES also contributes to poorer patient-reported outcomes [26] and increases the risk of early mortality [27] after hip replacement surgery. However, it is still unclear how the socioeconomic profile is reflected in the population that utilizes first-line treatment for hip and knee OA. The aim of the present study was to describe the SES of the population, who had been referred to an OA self-management program and registered in the BOA Register. The secondary aim was to evaluate if this population reflected the general population with regard to SES.

## Methods

### Study design

This is an observational register-based study with a cross-sectional design using data from a nationwide OA population registered in the Swedish BOA Register. The patients were registered before participation in an OA self-management program. A matched cohort from the general Swedish population was used for comparison. This study was conducted following the Strengthening the Reporting of Observational Studies in Epidemiology (STROBE) recommendations [28].

### Setting and data sources

All patients from the BOA Register with a first registration between 2008 and 2016 (*n* = 75,482) were included in the study. BOA is a National Quality Register that covers all regions in Sweden. It was established in 2008, and includes patients who have sought treatment for hip and/or knee pain and who, after a confirmed OA diagnosis, (clinical and/or radiographic, according to internationally accepted guidelines for diagnostic criteria [29, 30]), have been referred to a structured OA self-management program described previously by Thorstensson et al. [5]. One exclusion criterion for registration in the BOA Register and participation in the program is the presence of another disease that causes more severe problems than OA, such as suspicion of or confirmed tumor, inflammatory joint disease, sequelae of hip fracture or chronic widespread pain. Other exclusion criteria are total joint replacement within the previous 12 months, other surgery of the knee or hip joint within the previous 3 months and inability to read or understand Swedish [5]. In the present study, the BOA Register was used to identify registered individuals before their participation (baseline) in the OA self-management program. Data on most affected joint (hip or knee) was extracted, but no other patient data were extracted from the register, including data about compliance with the program.

**Fig. 1:**
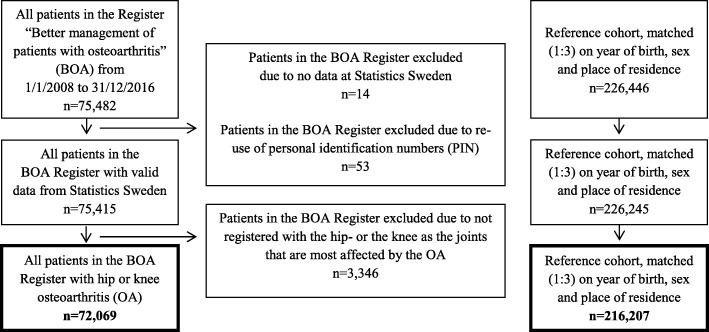
Flowchart of the study design

Without involvement from the researchers in the present study, a reference cohort (*n* = 226,446) was randomly selected by the government agency Statistics Sweden, from the Swedish Total Population Register (TPR) through direct matching for year of birth, sex and place of residence (geographical regions in Sweden, *n* = 21) at baseline (Fig. 1). All individuals were identified by their unique 10-digit personal identity number (PIN) that is assigned to all Swedish residents at birth or at immigration [31]. The TPR is often used for selection of general population controls in register-based research, due to the high quality, timeliness and the completeness (close to 100%) of the register and the possibility to identify individuals by the PIN [32]. To increase statistical power, three reference individuals were identified for each patient in the BOA population. Those individuals had never been included in the BOA Register.

Individual-level data on socioeconomic factors were obtained from two registers governed by Statistics Sweden; the TPR [32] which provides data on life events such as births, deaths, place of residence and marital status and the Longitudinal Integration Database for Health Insurance and Labour Market Studies (LISA) which provides other socioeconomic data such as educational level, occupation and disposable income [33]. Data were collected from Statistics Sweden at baseline in BOA for both populations, meaning that, for example, if the baseline time point for a patient in the BOA population was 2010, SES data were obtained for 2010 for that individual and for his or her matched reference individuals. The linkage between the registers and the merging of data was based on the individual-unique PIN [31]. More details can be found in an additional file and in the study protocol [34] (see Additional file 1).

Because of delays in the compilation of data at Statistics Sweden, data from LISA for both the BOA and the reference cohorts with baseline during 2016 were not available. In the analyses of variables from LISA (family type, educational level, employment, occupation, disposable income and sick leave), only individuals with baseline year between 2008 and 2015 were analyzed (except for the variable educational level, see below). The data collected from TPR (residential municipality, country of birth, marital status) included data from 2016.

### Socioeconomic indicators

Nine indicators of SES were used to describe the BOA population and compare it with the general Swedish population: 1) residential municipality, 2) country of birth, 3) marital status, 4) family type, 5) educational level, 6) employment, 7) occupation, 8) disposable income and 9) sick leave. These indicators were chosen because they have been shown previously to be SES factors associated with both disease burden and long-term outcome in OA [12, 25, 26, 35]. All indicators are described in detail in the additional file, including the source of the data and a description of the analyzed population (see Additional file 1). Briefly, they were defined as follows:

1. Residential municipalities (*n* = 290 in Sweden) were categorized as urban, suburban or rural.
2. Country of birth was categorized as Sweden, the Nordic countries (except Sweden), Europe (except the Nordic countries) or other countries.
3. Marital status was categorized as married (including registered partner) or not married.
4. Data on family type was categorized as cohabitation or not cohabitation, to capture a clearer measurement of the social support for individuals living together whether or not they were married.
5. The highest achieved educational level was classified by Statistics Sweden according to the Swedish Educational Terminology (SUN2000). For this study, we converted the classification into three categories: low (≤primary school [0–9 years]), medium (secondary school plus up to < 3 years postsecondary education [10–14 years]) and high (postsecondary education ≥3 years [≥15 years]). For the participants with baselines in 2016, the highest achieved level at 2015 was used because in these populations, education can be considered as a stable variable that does not change.
6. Employment was categorized as employed (including self-employed) or unemployed. For those > 65 years, the description “retired” was used if they were not registered as employed, because the general age of retirement in Sweden is 65 years.
7. Occupation was analyzed among those aged ≤65 years who were categorized as employed at baseline. Occupation was categorized as white-collar workers (non-manual labor) and blue-collar workers (manual labor) (see Additional file 1). This classification was used to distinguish occupations with lower or higher physical demands that also tend to include individuals who have higher or lower SES, respectively.
8. The household’s total disposable income (including e.g. income from employments, social welfare, pension, sickness benefits, minus taxes and deductions. For detail, see Additional file 1) was used to describe the individual’s disposable income. To be able to make comparisons of disposable income between different types of households, Statistics Sweden has calculated a weighting system related to the composition of the household (see Additional file 1). Data for disposable income were gathered for the baseline year and the 3 years prior to that year; however, for this study, disposable income was only reported during the baseline year (see comments in Additional file 1). The household disposable income was also categorized into quartiles, which were calculated by using the mean disposable income over the period (baseline year and 3 years prior) for the reference cohort, because this reflected the general Swedish population. The quartiles were created to compare the different study populations (e.g., those with knee or hip OA and their reference individuals) with each other. When reporting the results in this study, disposable income is expressed in Euros (€). During January to September 2019, the average value was: €1.00 = 10.57 Swedish kronor (SEK) [36].
9. Data about sick leave was collected for the baseline year and 3 years prior to that year (each year separate) in the form of the number of days of sick leave that exceeded 15 (see Additional file 1). In Sweden it is possible to be entitled to partial sick leave. This study focused on the duration of the sick leave, so that one day of sick leave was classified as one calendar day (gross day) regardless of whether it was part-time or full-time sick leave. Because information about the cause of the sick leave was not available, all days of sick leave of an individual was reported, not only those due to OA.

### Statistical analyses

Analyses were performed on groups of the BOA population based on whether they were registered as having OA that most affected their hip or knee joint. In case more than one joint was reported as affected by OA, only the joint with the most severe symptoms (according to the physiotherapist) was considered for the analysis. The populations with hip OA and knee OA were then compared with their respective matched reference groups. The populations were also analyzed separately grouped by age (≤65 years or > 65 years, because the general age of retirement in Sweden is 65 years), by sex and by type of work (white- or blue-collar workers).

Frequencies and percentages were used to describe categorical variables, including the 95% confidence interval (CI) for group comparison of proportions. Means, standard deviations (SD), median and interquartile range (IQR) were calculated for continuous variables.

No data were reported from Statistics Sweden for individuals who had died during the baseline year. Because the SES of these individuals was assumed to be lower and the distribution between the populations was unequal (BOA population 0.015‰ and reference cohort 0.09‰), the choice was made not to exclude these individuals but to impute the last known data for each variable. For example if an individual had died during the year 2015, data on disposable income during 2014 was used.

The merging of data from Statistics Sweden and the creation of the database was performed using SAS 9.4 TS Level 1MS. All statistical analyses were performed with IBM SPSS Statistics for Windows, v25.0 (IBM Statistics, Armonk, NY).

## Results

### Description of populations

In total, 72,069 individuals from the BOA Register, of whom 32% had hip OA (*n* = 22,703) and 68% had knee OA (*n* = 49,366), were included in the study. In addition, a reference cohort of 216,207 individuals from the general population in Sweden was generated: 68,109 to match the individuals of the BOA population with hip OA and 148,098 to match those with knee OA (Fig. 1).

### The BOA population with hip OA

The BOA population with hip OA had consistently higher SES than their reference group (Table 1). More of them were born in Sweden and more of them were married, cohabiting and employed, than in their reference group. This BOA population had achieved a higher level of education, had on average a higher disposable income and a higher proportion had days of sick leave during their baseline year than those in their reference group. These differences between the BOA and the reference group remained when analyses were performed separately for those aged ≤65 years and > 65 years, respectively.

**Table 1:** Socioeconomic indicators for the BOA populations with hip OA, grouped by age

	**Hip OA total**		**Hip OA ≤65**		**Hip OA > 65 years**	
	Hip OA	Reference group	Hip OA	Reference group	Hip OA	Reference group
***Population***	*n = 22,703*	*n = 68,109*	*n = 8733*	*n = 26,199*	*n = 13,970*	*n = 41,910*
**Women**	68 (67.4, 68.6)	68 (67.6, 68.4)	68 (67.0, 69.0)	68 (67.4, 68.6)	68 (67.2, 68.8)	68 (67.6, 68.4)
**Age in years** *	67.1 (9.6)	67.1 (9.6)	57.4 (6.5)	57.4 (6.5)	73.2 (5.3)	73.2 (5.3)
*Missing*	*n = 0*	*n = 0*	*n = 0*	*n = 0*	*n = 0*	*n = 0*
**Urban/suburban living**	69 (68.4, 69.6)	68 (67.6, 68.4)	69 (68.0, 69.0)	68 (67.4, 68.6)	69 (68.2, 69.8)	67 (66.5, 67.5)
*Missing*	*n = 5*	*n = 48*	*n = 3*	*n = 29*	*n = 2*	*n = 19*
**Married**	58 (57.4, 58.6)	54 (53.6, 54.4)	57 (56.0, 58.0)	55 (54.4, 55.6)	59 (58.2, 59.8)	54 (53.5, 54.5)
*Missing*	*n = 5*	*n = 49*	*n = 3*	*n = 30*	*n = 2*	*n = 19*
**Born in Sweden**	92.5 (92.2, 92.8)	87 (86.7, 87.3)	91.5 (90.9, 92.1)	84.5 (84.1, 84.9)	93 (92.6, 93.4)	88.5 (88.2, 88.8)
The Nordic countries	4 (3.7, 4.3)	5 (4.8, 5.2)	3.5 (3.1, 3.9)	4 (3.8, 4.2)	4 (3.7, 4.3)	5.5 (5.3, 5.7)
Europe	2.5 (2.3, 2.7)	4.5 (4.3, 4.7)	3 (2.6, 3.4)	5.5 (5.2, 5.8)	2.5 (2.2, 2.8)	4 (3.8, 4.2)
Other countries	1 (0.9, 1.1)	3.5 (3.4, 3.6)	2 (1.7, 2.3)	6 (5.7, 6.3)	0.5 (0.4, 0.6)	2 (1.9, 2.1)
*Missing*	*n = 0*	*n = 3*	*n = 0*	*n = 0*	*n = 0*	*n = 3*
**Educational level**						
Low (0–9 years)	22.5 (22.0, 23.0)	30 (29.7, 30.3)	13 (12.3, 13.7)	18 (17.5, 18.5)	28 (27.3, 28.7)	37 (36.5, 37.5)
Medium (10–14 years)	59.5 (58.9, 60.1)	55 (54.6, 55.4)	69 (68.0, 70.0)	64 (63.4, 64,6)	54 (53.2, 54.8)	50 (49.5, 50.5)
High (≥15 years)	18 (17.5, 18.5)	15 (14.7, 15.3)	18 (17.2, 18.8)	18 (17.5, 18.5)	17 (16.4, 17.6)	13 (12.7, 13.3)
*Missing*	*n = 65*	*n = 762*	*n = 16*	*n = 213*	*n = 49*	*n = 549*
***Population*** ^**a**^	*n = 17,153*	*n = 51,459*	*n = 6753*	*n = 20,259*	*n = 10,400*	*n = 31,200*
**Cohabitation**	64 (63.3, 64.7)	60 (59.6, 60.4)	69 (67.9, 70.1)	66 (65.3, 66.7)	61 (60.1, 61.9)	56 (55.4, 56.6)
*Missing*	*n = 2*	*n = 46*	*n = 1*	*n = 28*	*n = 1*	*n = 18*
**Employed**	36 (35.3, 36.7)	34 (33.6, 34.4)	75 (74.0, 76.0)	71 (70.4, 71.6)	11 (10.4, 11.6)	10 (9.7, 10.3)
*Missing*	*n = 5*	*n = 83*	*n = 2*	*n = 34*	*n = 3*	*n = 49*
**Sick leave** ^**b**^	10 (9.6, 10.4)	5 (4.8, 5.2)	26 (25.0, 27.0)	13 (12.5, 13.5)	0.003 (−0.008, 0.014)	0.002 (−0.003, 0.007)
Days of sick leave **	78 (170)	64 (163)	80 (170)	66 (167)	39 (43)	41 (108)
*Missing*	*n = 2*	*n = 46*	*n = 1*	*n = 28*	*n = 1*	*n = 18*
**Income quartile** ^**b**^						
Lowest (≤12,262)	17 (16.4, 17.6)	26 (25.6, 26.4)	12 (11.2, 12.8)	18 (17.5, 18.5)	21 (20.2, 21.8)	32 (31.5, 32.5)
2nd (12,263-16,654)	28 (27.3, 28.7)	27 (26.6, 27.4)	17 (16.1, 17.9)	17 (16.5, 17.5)	36 (35.1, 36.9)	33 (32.5, 33.5)
3rd (16,655-22,962)	27 (26.3, 27.7)	23 (22.6, 23.4)	32 (30.9, 33.1)	28 (27.4, 28.6)	23 (22.2, 23.8)	19 (18.6, 19.4)
Highest (≥22,963)	28 (27.3, 28.7)	24 (23.6, 24.4)	39 (37.8, 40.2)	37 (36.3, 37.7)	20 (19.2, 20.8)	16 (15.6, 16.4)
**Income** ^**c**^ **	17,442 (10, 478)	15,998 (10,659)	20,610 (10,512)	19,931 (11,979)	15,533 (8531)	14,165 (7783)
*Missing*	*n = 2*	*n = 46*	*n = 1*	*n = 28*	*n = 1*	*n = 18*
**Occupation** ^**d**^						
White-collar workers			54 (52.5, 55.5)	55 (54.1, 55.9)		
% with sick leave ^b^			24 (22.3, 25.7)	12 (11.2, 12.8)		
Days of sick leave **			55 (131)	53 (132)		
Blue-collar workers			46 (44.5, 47.5)	45 (44.1, 45.7)		
% with sick leave ^b^			37 (34.9, 39.1)	17 (16.0, 18.0)		
Days of sick leave **			78 (152)	41 (104)		
*Missing*			*n = 642*	*n = 1757*		

Description of socioeconomic indicators for the BOA population with hip OA and their reference groups from the general Swedish population, in total and grouped by age. Percentages and confidence interval (CI upper, lower) is reported, if no other information is given. *Mean (SD). **Median (IQR)

^a^The variables below were only analyzed among those individuals with their baseline year between 2008 and 2015

^b^During the baseline year

^c^The amount is stated in Euro (€)

^d^Among those that were ≤ 65 years and categorized as employed at baseline

**Fig. 2:**
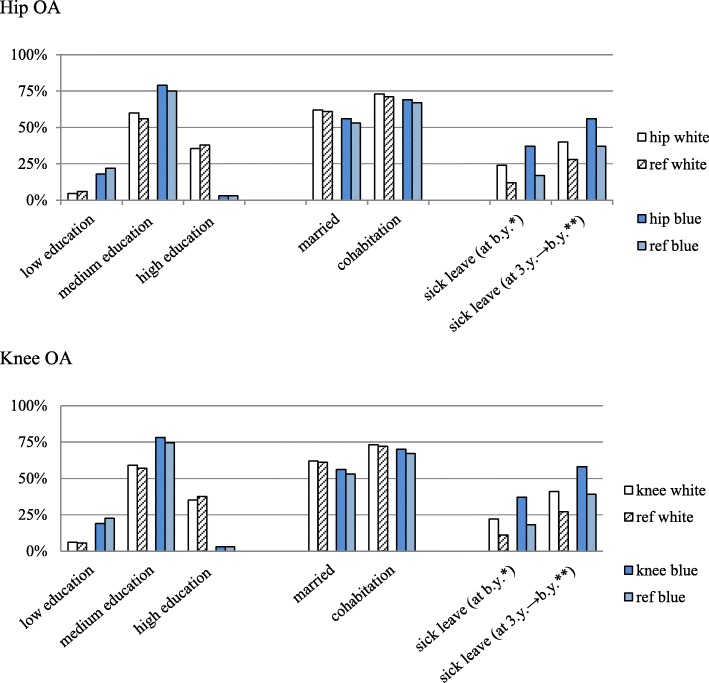
Socioeconomic indicators for the BOA population, grouped by most affected joint and occupation. Description of education, marital status, family type and sick-leave for the BOA population compared with their reference groups from the general Swedish population. Those who were aged ≤65 years, categorized as employed and registered with an occupation (white- or blue-collar work) at baseline were analyzed. *Sick leave during baseline year. **Sick leave during baseline year and 3 years prior to that year

When individuals with hip OA who were aged ≤65 years, employed and registered with an occupation (white- or blue-collar work) were analyzed separately, the educational level, marital status and rate of cohabitation were similar in the BOA and reference groups (Fig. 2). Disposable income during the baseline year was a median (IQR) of €24,463 (11,072) in the white-collar workers in the BOA population compared with €24,577 (11,106) for their reference group. For blue-collar workers, disposable income in the BOA population was €19,732 (8127) compared with €19,684 (8588) in their reference group during the same period. Among white-collar workers, a higher proportion of the BOA population with hip OA had days of sick leave during their baseline year compared with their reference group (24% vs 12%). Among blue-collar workers the distribution was 37% vs 17%. When we compared sick leave in the BOA population during their baseline year and 3 years prior to that year with that in the reference group, 40% vs 28% of the white-collar workers in the BOA and reference groups, respectively, and 56% vs 37% of the blue-collar workers in the BOA and reference groups, respectively, had days of sick leave (Fig. 2).

### The BOA population with knee OA

Similarly to the hip OA population, the BOA population with knee OA had a higher SES than their reference group (Table 2). More of them were born in Sweden and more of them were married, cohabiting and employed compared with their reference group. This population had achieved a higher level of education, had on average a higher disposable income and a higher proportion had days of sick leave during their baseline year compared with their reference group. When those aged ≤65 years and > 65 years were analyzed separately, the differences in disposable income remained, but the differences in educational level were only present among those aged > 65 years. A higher proportion of the BOA population aged ≤65 years was employed, while among those aged > 65 years, the proportion of retirees was similar in the BOA population and their reference group.

**Table 2:** Socioeconomic indicators for the BOA populations with knee OA, grouped by age

	**Knee OA total**		**Knee OA ≤65 years**		**Knee OA > 65 years**	
	Knee OA	Reference group	Knee OA	Reference group	Knee OA	Reference group
***Population***	*n = 49,366*	*n = 148,098*	*n = 22,075*	*n = 66,225*	*n = 27,291*	*n = 81, 873*
**Women**	69 (68.6, 69.4)	69 (68.8, 69.2)	71 (70.4, 71.6)	71 (70.7, 71.3)	67.5 (66.9, 68.1)	67.5 (67.2, 67.8)
**Age in years** *	66.1 (9.6)	66.1 (9.6)	57.6 (6.2)	57.6 (6.2)	73.0 (5.3)	73.0 (5.3)
*Missing*	*n = 0*	*n = 0*	*n = 0*	*n = 0*	*n = 0*	*n = 0*
**Urban/suburban living**	70 (69.6, 70.4)	68 (67.8, 68.2)	69 (68.4, 69.6)	68 (67.6, 68.4)	70 (69.5, 70.5)	68 (67.7, 68.3)
*Missing*	*n = 4*	*n = 112*	*n = 2*	*n = 71*	*n = 2*	*n = 41*
**Married**	59 (58.6, 59.4)	55 (54.7, 55.3)	58 (57. 3, 58.7)	55 (54.6, 55.4)	60 (59.4, 60.6)	54 (53.7, 54.3)
*Missing*	*n = 4*	*n = 113*	*n = 2*	*n = 71*	*n = 2*	*n = 42*
**Born in Sweden**	90 (89.7, 90.3)	87 (86.8, 87.2)	88 (87.6, 88.4)	84.5 (84.2, 84.8)	92 (91.7, 92.3)	88 (87.8, 88.2)
The Nordic countries	4 (3.8, 4.2)	4.5 (4.4, 4.6)	4 (3.7, 4.3)	4 (3.9, 4.1)	4 (3.8, 4.2)	5.5 (5.3, 5.7)
Europe	3.5 (3.3, 3.7)	5 (4.9, 5.1)	4 (3.7, 4.3)	6 (5.8, 6.2)	3 (2.8, 3.2)	4.5 (4.4, 4.6)
Other countries	2.5 (2.4, 2.6)	3.5 (3.4, 3.6)	4 (3.7, 4.3)	5.5 (5.3, 5.7)	1 (0,9, 1.1)	2 (1.9, 2.1)
*Missing*	*n = 0*	*n = 9*	*n = 0*	*n = 5*	*n = 0*	*n = 4*
**Educational level**						
Low (0–9 years)	23 (22.6, 23.4)	28 (27.8, 28.2)	15 (14.5, 15.5)	17.5 (17.2, 17.8)	29 (28.5, 29.5)	37 (36.7, 37.3)
Medium (10–14 years)	61 (60.6, 61.4)	56 (55.7, 56.3)	69 (68.4, 69.6)	63.5 (63.1, 63.9)	54 (53.4, 54.6)	50 (49.7, 50.3)
High (≥15 years)	16 (15.7, 16.3)	16 (15.8, 16.2)	16 (15.5, 16.5)	19 (18.7, 19.3)	17 (16.6, 17.4)	13 (12.8, 13.2)
*Missing*	*n = 125*	*n = 1557*	*n = 26*	*n = 492*	*n = 99*	*n = 1065*
***Population*** ^**a**^	*n = 37,940*	*n = 113,820*	*n = 17,186*	*n = 51,558*	*n = 20,754*	*n = 62,262*
**Cohabitation**	65 (64.5, 65.5)	60 (59.7, 60.3)	70 (69.3, 70.7)	66 (65.6, 66.4)	62 (61.3, 62.7)	56 (55.6, 56.4)
*Missing*	*n = 2*	*n = 86*	*n = 2*	*n = 57*	*n = 0*	*n = 29*
**Employed**	40 (39.5, 40.5)	38 (37.7, 38.3)	75 (74.4, 75.6)	72 (71.6, 72.4)	11 (10.6, 11.4)	11 (10.8, 11.2)
*Missing*	*n = 6*	*n = 162*	*n = 2*	*n = 69*	*n = 4*	*n = 93*
**Sick leave** ^**b**^	12 (11.7, 12.3)	6 (5.9, 6.1)	26 (25.3, 26.7)	12 (11.7, 12.3)	0.004 (−0.005, 0.013)	0.003 (−0.001, 0.007)
Days of sick leave **	71 (159)	59 (164)	72 (161)	59 (165)	52 (55)	33 (88)
*Missing*	*n = 2*	*n = 86*	*n = 2*	*n = 57*	*n = 0*	*n = 29*
**Income quartile** ^**b**^						
Lowest (≤12,262)	17 (16.6, 17.4)	25 (24.8, 25.3)	12 (11.5, 12.5)	17 (16.7, 17.3)	21 (20.5, 21.6)	31 (30.6, 31.4)
2nd (12,263-16,654)	27 (26.6, 27.4)	25.5 (25.3, 25.8)	18 (17.4, 18.6)	16.5 (16.2, 16.8)	35 (34.4, 35.7)	33 (32.6, 33.4)
3rd (16,655-22,9624)	28 (27.6, 28.5)	23 (22.8, 23.2)	32 (31.3, 32.7)	29 (28.6, 29.4)	24 (23.4, 24.6)	19 (18.7, 19.3)
Highest (≥22,963)	28 (27.5, 28.5)	26.5 (26.2, 26.8)	38 (37.3, 38.7)	37.5 (37.1, 37.9)	20 (19.5, 20.5)	17 (16.7, 17.3)
**Income** ^**c**^ **	17,794 (10,574)	16,578 (11,221)	20,511 (10,410)	20,131 (11,913)	15,637 (8515)	14,269 (7942)
*Missing*	*n = 2*	*n = 86*	*n = 2*	*n = 57*	*n = 0*	*n = 29*
**Occupation** ^**d**^						
White-collar workers			49 (48.1, 50.0)	56 (55.5, 56.5)		
% with sick leave ^b^			22 (20.9, 23.1)	11 (10.5, 11.5)		
Days of sick leave **			49 (119)	45 (122)		
Blue-collar workers			51 (50.1, 51.9)	44 (43.5, 44.5)		
% with sick leave ^b^			37 (35.8, 38.2)	18 (17.6, 18.6)		
Days of sick leave **			59 (130)	41 (107)		
*Missing*			*n = 1569*	*n = 4447*		

Description of socioeconomic indicators for the BOA population with knee OA and their reference groups from the general Swedish population, in total and grouped by age. Percentages and confidence interval (CI upper, lower) is reported, if no other information is given. *Mean (SD). **Median (IQR)

^a^The variables below were only analyzed among those individuals with their baseline year between 2008 and 2015

^b^During the baseline year

^c^The amount is stated in Euro (€)

^d^Among those that were ≤ 65 years and categorized as employed at baseline

When individuals with knee OA who were aged ≤65 years, employed and registered with an occupation (white- or blue-collar work) were analyzed separately, the educational level, marital status and rate of cohabitation were similar in the BOA and reference groups (Fig. 2). Disposable income during the baseline year was a median (IQR) €24,292 (10,574) for white-collar workers in the BOA population compared with €24,795 (11,656) in their reference group. For blue-collar workers, disposable income in the BOA population was €19,817 (8182) compared with €19,751 (8217) in the reference group during the same period. Among white-collar workers, a higher proportion of the BOA population with knee OA had days of sick leave during their baseline year compared with their reference group (22% vs 11%). Among blue-collar workers the distribution was 37% vs 18%. When sick leave during the year of baseline and 3 years prior to that year was analyzed, 41% vs 27% of the white-collar workers and 58% vs 39% of the blue-collar workers in the BOA and reference group, respectively, had days of sick leave (Fig. 2).

### Socioeconomic indicators by sex

When the populations were analyzed by sex, the differences between the BOA populations with hip and knee OA and their reference groups were similar to those seen for the total population. Women consistently had lower SES than men in both the BOA and reference groups, except for educational level: a higher proportion of the women had achieved a higher level of education. A higher proportion of women than men in both the BOA population and in the reference group had days of sick leave (Table 3).

**Table 3:** Socioeconomic indicators for the BOA populations with hip OA, grouped by sex

	**Hip OA male (32%)**		**Hip OA female (68%)**		**Knee OA male (31%)**		**Knee OA female (69%)**	
	Hip OA	Reference group	Hip OA	Reference group	Knee OA	Reference group	Knee OA	Reference group
***Population***	*n = 7293*	*n = 21,879*	*n = 15,410*	*n = 46,230*	*n = 15,282*	*n = 45,846*	*n = 34,084*	*n = 102,252*
Age in years *	67.2 (9.7)	67.2 (9.7)	67.1 (9.6)	67.1 (9.6)	66.7 (9.5)	66.7 (9.5)	65.8 (9.6)	65.8 (9.6)
*Missing*	*n = 0*	*n = 0*	*n = 0*	*n = 0*	*n = 0*	*n = 0*	*n = 0*	*n = 0*
**Urban/suburban living**	68 (66.9, 69.1)	67 (66.4, 67.6)	69 (68.3, 69.7)	68 (67.6, 68.4)	68 (67.3, 68.7)	67 (66.6, 67.4)	70 (69.5, 70.5)	69 (68.7, 69.3)
*Missing*	*n = 1*	*n = 19*	*n = 4*	*n = 29*	*n = 3*	*n = 42*	*n = 1*	*n = 70*
**Married**	65.5 (64.4, 66.6)	60 (59.4, 60.6)	55 (54.2, 55.8)	52 (51.5, 52.5)	67 (66.3, 67.7)	60 (59.6, 60.4)	56 (55.5, 56.5)	52 (51.7, 52.3)
*Missing*	*n = 1*	*n = 20*	*n = 4*	*n = 29*	*n = 3*	*n = 42*	*n = 1*	*n = 71*
**Born in Sweden**	94 (93.5, 94.5)	88 (87.6, 88.4)	92 (91.6, 92.4)	87 (86.7, 87.3)	92 (91.6, 92.4)	88 (87.7, 88.3)	89 (88.7, 89.3)	86 (85.8, 86.2)
The Nordic countries	3 (2.6, 3.4)	4 (3.7, 4.3)	4 (3.7, 4.3)	5 (4.8, 5.2)	3 (2.7, 3.3)	4 (3.8, 4.2)	4 (3.8, 4.2)	5 (4.9, 5.1)
Europe	2 (1.7, 2.3)	4.5 (4.2, 4.8)	3 (2.7, 3.3)	5 (4.8, 5.2)	3 (2.7, 3.3)	4.5 (4.3, 4.7)	4 (3.8, 4.2)	5 (4.9, 5.1)
Other countries	1 (0,8, 1.2)	3.5 (3.3, 3.7)	1 (0.8, 1.2)	3 (2.8, 3.2)	2 (1.8, 2.2)	3.5 (3.3, 3.7)	3 (2.8, 3.2)	4 (3.9, 4.1)
*Missing*	*n = 0*	*n = 2*	*n = 0*	*n = 1*	*n = 0*	*n = 2*	*n = 0*	*n = 7*
**Educational level**								
Low (0–9 years)	26.5 (25.5, 27.5)	32.5 (31.9, 33.1)	21 (20.4, 21.6)	28 (27.6, 28.4)	26.5 (25.8, 27.2)	32 (31.8, 32.4)	21 (20.6, 21.4)	26.5 (26.2, 26.8)
Medium (10–14 years)	58 (56.9, 59.1)	54 (53.3, 54.7)	60 (59.2, 60.8)	56 (55.5, 56.5)	60 (59.2, 60.8)	54 (53.5, 54.5)	61 (60.5, 61.5)	57 (56.7, 57.3)
High (≥15 years)	15.5 (14.7, 16.3)	13.5 (13.0, 14.0)	19 (18.4, 19.6)	16 (15.7, 16.3)	13.5 (13.0, 14.0)	14 (13.7, 14.3)	18 (17.6, 18.4)	16.5 (16.3, 16.7)
*Missing*	*n = 18*	*n = 236*	*n = 47*	*n = 526*	*n = 51*	*n = 446*	*n = 74*	*n = 1111*
***Population*** ^***a***^	*n = 5457*	*n = 16,371*	*n = 11,696*	*n = 35,088*	*n = 11,519*	*n = 34,557*	*n = 26,421*	*n = 79,263*
**Cohabitation**	72 (70.8, 73.2)	65 (64.3, 65.7)	61 (60.1, 61.9)	57 (56.5, 57.5)	72 (71.2, 72.8)	65 (64.5, 65.5)	62 (61.4, 62.6)	58 (57.7, 58.3)
*Missing*	*n = 1*	*n = 18*	*n = 1*	*n = 28*	*n = 2*	*n = 27*	*n = 0*	*n = 59*
**Employed**	40 (38.7, 41.3)	38 (37.3, 38.7)	34 (33.1, 34.9)	32 (31.5, 32.5)	43 (42.1, 43.9)	40.5 (40.0, 41.0)	39 (38.4, 39.6)	38 (37.7, 38.3)
*Missing*	*n = 4*	*n = 32*	*n = 1*	*n = 51*	*n = 6*	*n = 53*	*n = 0*	*n = 109*
**Sick leave** ^**b, c**^	24 (22.2, 25.8)	10 (9.3, 10.7)	27 (25.7, 28.3)	14 (13.4, 14.6)	23 (21.8, 24.2)	9 (8.5, 9.5)	27 (26.2, 27.8)	13 (12.7, 13.3)
Days of sick leave **	78 (163)	70 (164)	80 (175)	64 (169)	69 (157)	56 (156)	73 (161)	59 (167)
*Missing*	*n = 0*	*n = 11*	*n = 1*	*n = 17*	*n = 2*	*n = 20*	*n = 0*	*n = 37*
**Income quartile** ^**c**^								
Lowest (≤12,262)	13 (12.1, 13.9)	22 (21.4, 22.6)	19.5 (18.8, 20.2)	28.5 (28.0, 29.0)	12 (11.4, 12.6)	21 (20.6, 21.4)	19 (18.5, 19.5)	27 (26.7, 27.3)
2nd (12,263-16,654)	27 (25.8, 28.2)	27 (26.3, 27.7)	29 (28.2, 29.8)	26.5 (26.0, 27.0)	26 (25.2, 26.8)	26 (25.5, 26.5)	28 (27.5, 28.5)	25 (24.7, 25.3)
3rd (16,655-22,962)	29 (27.8, 30.2)	24 (23.3, 24.7)	25.5 (24.7, 26.3)	22 (21.6, 22.4)	30 (29.2, 30.8)	24 (23.5, 24.5)	27 (26.5, 27.5)	23 (22.7, 23.3)
Highest (≥22,963)	31 (29.8, 32.2)	27 (26.3, 27.7)	26 (25.2, 26.8)	23 (22.6, 23.4)	32 (31.2, 32.9)	29 (28.5, 29.5)	26 (25.5, 26.5)	25 (24.7, 25.3)
**Income** ^***d***^ **	18,544 (10,754)	16,948 (10,991)	16,977 (10,355)	15,552 (10,379)	19,000 (10,631)	17,366 (11,410)	17,243 (10,384)	16,236 (11,058)
*Missing*	*n = 1*	*n = 18*	*n = 1*	*n = 28*	*n = 2*	*n = 27*	*n = 0*	*n = 59*
**Occupation**^**e**^								
White-collar workers	52 (49.4, 54.6)	50 (48.4, 51.6)	54 (52.2, 55.8)	57 (56.0, 58.0)	44 (42.3, 45.7)	50 (49.0, 51.0)	51 (49.9, 52.1)	58 (57.4, 58.6)
% with sick leave ^b, c^	19 (16.2, 21.8)	8 (6.8, 9.2)	26 (23.9, 28.1)	14 (13.0, 15.0)	16 (14.1, 17.9)	7 (6.3, 7.7)	24 (22.7, 25.3)	13 (12.4, 13.6)
Days of sick leave **	50 (118)	59 (144)	59 (133)	51 (130)	49 (111)	51 (124)	50 (122)	44 (121)
Blue-collar workers	48 (45.4, 50.6)	50 (48.4, 51.6)	46 (44.2, 47.8)	43 (42.0, 44.0)	56 (54.3, 57.7)	50 (49.0, 51.0)	49 (47.9, 50.1)	42 (41.4, 42.6)
% with sick leave ^c^	33 (29.5, 36.5)	12 (10.6, 13.4)	39 (36.4, 41.6)	19 (17.7, 20.3)	32 (29.9, 34.1)	14 (13.0, 15.0)	40 (38.5, 41.5)	20 (19.2, 20.8)
Days of sick leave **	78 (144)	35 (90)	77 (155)	42 (108)	55 (120)	37 (100)	61 (135)	42 (109)
*Missing*	*n = 257*	*n = 746*	*n = 385*	*n = 1011*	*n = 657*	*n = 1855*	*n = 912*	*n = 2592*

Description of socioeconomic indicators for the BOA populations with hip OA compared to their reference groups from the general Swedish population, grouped by sex

Percentages and confidence interval (CI upper, lower) is reported, if no other information is given. *Mean (SD). **Median (IQR)

^a^The variables below were only analyzed among those individuals with their baseline year between 2008 and 2015

^b^Among those ≤65 years

^c^During the baseline year. ^d^ The amount is stated in Euro (€)

^e^Among those that were ≤ 65 years and categorized as employed at baseline

## Discussion

This study shows that patients in the BOA population who have hip or knee OA, had an overall higher SES than the general population. A higher proportion of them were born in Sweden, married, cohabiting and employed. The BOA population had also achieved a higher level of education and had a higher disposable income compared with the reference cohort from the general population. Similar results were found for nearly all of the SES indicators when the populations were analyzed by age and sex. In contrast, the BOA population had more days of sick leave than the reference cohort.

Previous research has shown that OA of hip [21] and knee [19] is more common in people with lower educational attainment compared to people with high educational level. It has also been reported that those with lower education have reduced access to self-management programs as treatment for OA [25]. The results of the present study showed that higher educational attainment was more common in the BOA population, indicating that, also in Sweden, self-management programs for OA may have difficulty reaching more socioeconomically disadvantaged groups of individuals, in accordance with previous studies. These individuals are also potentially those in most need of supportive interventions because they often have a higher disease burden from OA [15, 21–23].

Educational level is considered the most stable indicator of SES and as an indicator of SES in early adult life, before chronic age-related diseases such as OA have occurred [20]. It is also a strong determinant of future income and choice of occupation, because it influences which jobs are available. In contrast, income is the SES indicator that most directly measures material resources and can change the most over a short period [15]. When we analyzed disposable income in this study, the BOA population had on average a higher disposable income than the reference cohort. This difference remained when individuals were grouped by age or sex, but when we only analyzed those aged ≤65 years who had an occupation (white- or blue-collar work), the reference groups had a similar or higher disposable income than the BOA population. A likely explanation for the loss of income difference between groups in those aged ≤65 years is the higher prevalence of sick leave in the BOA population. The available data do not allow us to determine whether the sick leave was due to OA or other diseases, but because OA is associated with a higher prevalence of several other diseases, such as diabetes mellitus, cardiovascular diseases and depressive symptoms [37–39], the overall risk of sick leave in the BOA population is probably increased. In support, it has previously been shown that OA increases the risk of short and long periods of sick leave [40].

An interesting finding was the consistently lower SES among women compared with men, regardless of if they were in the BOA population or the reference cohort. These differences were detected in all SES indicators except educational level. This is however not exclusive for the populations that we studied, but more reflect the gender differences that unfortunately exists in society today.

### Limitations and strengths of the study

It is important to consider some factors in interpreting the results. First, because of difficulties in defining OA and identifying the onset of the disease [41], there were probably individuals with OA in the reference cohort. It is not possible to assess how or whether this affected the results of the present study. However, it is reasonable to assume that some individuals in a general population of this age group would have OA because the disease is very common.

Second, results from studies of diabetes and HIV [42], indicates that self-management treatments are better suited for individuals with higher compared to lower SES. To the best of our knowledge, this has not studied in OA yet, but could affect that individuals with lower SES are not included in the BOA Register to the same extend. The results from this study may also have been influenced by the fact that self-management programs such as BOA are less suitable for OA patients with otherwise poor health, who are also likely to have lower SES. However, we believe that this only explains a minor part of the differences identified between the BOA and reference cohorts. A more likely interpretation of the differences detected in the present study is that more disadvantaged individuals who suffers from chronic diseases such as OA are both less likely to seek healthcare [10] and also have less access to OA care [24, 25], and therefore are not included in the BOA Register today. Further research aims to clarify any differences in comorbidity between the BOA population and the general Swedish population.

During 2019, the BOA Register changed the criteria for registration to also include self-management program delivered as individual sessions or digital. Maybe also a comorbidity status and SES profile of OA patients would further help to achieve more equal treatment participation, personalized OA care and improved outcome.

The BOA Register is to the best of our knowledge, the largest database of collected information from a nationwide OA population that has been referred to a structured OA self-management program. The size of this population, together with the ability to merge data from the BOA Register with data from other Swedish sources of health and socioeconomic data provides the opportunity to study a range of factors that may influence the progression of OA and factors that can predict long-term outcomes of OA in the BOA population. In the present study, we focused on SES. The SES indicators that were evaluated may be related to each other. However, since we did not aim to explain or to evaluate the impact of a specific factor in this study, we chose only descriptive analyses of the SES indicators. With knowledge of the impact that SES has on both prevalence of OA and access to OA health care, it is important to first define and clarify the SES in the BOA population, to be able to determine the generalizability of this population in future studies.

## Conclusions

In conclusion, patients who have been referred to the national self-management program for hip and knee OA in Sweden had an overall higher SES than the general population. The results from this study indicate that this self-management program for OA may not reach the more socioeconomically disadvantaged groups, who are often those with a higher disease burden. To achieve equal health for all, SES should be considered when structuring healthcare systems.

## Supplementary information

**Additional file 1:** Description of the nine indicators of socioeconomic status (SES) used in the study.

## Data Availability

The dataset generated and/or analyzed during the current study is governed by Region Västra Götaland. The authors are not allowed to share the data. The data is available from Center of Registers Västra Götaland, Gothenburg, Sweden (contact: boa@registercentrum.se) for researchers who meet the criteria for access to confidential data according to Swedish law.

## Abbreviations

€: Euro
BOA: Better management of patients with Osteoarthritis
CI: Confidence interval
IQR: Interquartile range
LISA: The Longitudinal Integration Database for Health Insurance and Labour Market Studies
OA: Osteoarthritis
PIN: Personal identity number
SD: Standard deviations
SEK: Swedish kronor
SES: Socioeconomic status
TPR: The Swedish Total Population Register
