# Supplementary material for: Socioeconomic status of patients in a Swedish national self-management program for osteoarthritis compared with the general population—a descriptive observational study
Source: BMC Musculoskelet Disord. 2020 Jan 6;21:10. doi: 10.1186/s12891-019-3016-z (PMC6945568; doi:10.1186/s12891-019-3016-z)
Supplement: Supplementary file 1 — Additional file 1: Description of the nine indicators of socioeconomic status (SES) used in the study. [file 12891_2019_3016_MOESM1_ESM.docx]

Additional file 1: Description of the nine indicators of socioeconomic status (SES) used in the study

For each indicator in the study, a description of the following items is included:

- Source of data (which registry at Statistics Sweden)
- Type of data delivered from Statistics Sweden and its description
- Categories created for the purpose of this study
- Comments (as required)
- Data about the populations with baseline years

# 1) Residential municipality

## Source of data (which registry at Statistics Sweden)

The Swedish Total Population Register (TPR) at Statistics Sweden, which uses data from the Swedish Tax Agency’s Population Register (the national registration covering the whole Swedish population) [1].

## Type of data delivered from Statistics Sweden and its description

Statistics Sweden delivered data about each study participant’s residential municipality. There are 290 municipalities in Sweden. To classify residential municipality in this study, we used the Swedish Association of Local Authorities and Regions (SALAR) classification of Swedish municipalities, which is based on structural parameters such as population and commuting patterns, and contains three main categories that are divided into a total of nine subcategories [2].

## Categories created for the purpose of this study

In this study, residential municipality was classified according to the three main categories of the SALAR classification:

1. Urban: Large cities (≥200,000 inhabitants) and municipalities near large cities
2. Suburban: Medium-sized towns (≥40,000 inhabitants) and municipalities near medium-sized towns
3. Rural: Smaller towns/urban areas and rural municipalities

## Data about the populations with baseline years

Between 2008 and 2016

# 2) Country of birth

**Source of data (which registry at Statistics Sweden)**

TPR at Statistics Sweden, which uses data from the Swedish Tax Agency’s population register.

## Type of data delivered from Statistics Sweden and its description

Data on country of birth for each participant in the study was delivered in the following groups of countries. The figure in parentheses indicates how the data was categorized in this study.

- 00: Sweden (1)
- 01: The Nordic countries (except Sweden) (2)
- 02: EU28 except the Nordic countries (2)
- 03: Europe (except EU28 and the Nordic countries) (2)
- 04: Africa (2)
- 05: North America (2)
- 06: South America (2)
- 07: Asia (2)
- 08: Oceania (2)
- 09: The Soviet Union (2)
- 10: Unknown (2)

## Categories created for the purpose of this study

Country of birth was in this study categorized as:

1. Sweden
2. The Nordic countries (except Sweden)
3. Europe (except the Nordic countries)
4. Other countries

**Data about the populations with baseline years**

Between 2008 and 2016

# 3) Marital status

## Source of data (which registry at Statistics Sweden)

TPR at Statistics Sweden, which uses data from the Swedish Tax Agency’s population register.

## Type of data delivered from Statistics Sweden and its description

Data on marital status for each participant in the study was delivered in seven different categories. The figure in parentheses indicates how the data was categorized in this study.

- - Married (1)
  - Unmarried (2)
  - Divorced (2)
  - Widow/widower (2)
  - Registered partner (1)
  - Divorced partner (2)
  - Survival partner (2)

## Categories created for the purpose of this study

Marital status was in this study categorized as:

1. Married
2. Not married

## Data about the populations with baseline years

Between 2008 and 2016

# 4) Family type

## Source of data (which registry at Statistics Sweden)

The Longitudinal Integration Database for Health Insurance and Labour Market Studies (LISA) at Statistics Sweden, which derives data from the Swedish Tax Agency’s population register and links data between different individuals to create this variable.

## Type of data delivered from Statistics Sweden and its description

Data about family type for each participant in the study was delivered in 12 different categories. The figure in parentheses indicates how the data was categorized in this study.

- 11: Legally married and living together, including registered partnership (without children) (1)
- 12: Legally married and living together (including registered partnership) (with at least 1 child <18 years living at home) (1)
- 13: Legally married and living together (including registered partnership) (with at least 1 child ≥18 years still living at home) (1)
- 21: Consensual union partners (who have no children together) (1)
- 22: Consensual union partners (with at least 1 child <18 years living at home) (1)
- 23: Consensual union partners (with at least 1 child ≥18 years still living at home) (1)
- 31: Single father (with at least 1 child <18 years still living at home) (2)
- 32: Single father (youngest child living at home ≥18 years) (1)
- 41: Single mother (with at least 1 child <18 years still living at home) (2)
- 42: Single mother (youngest child living at home ≥18 years) (1)
- 50: Single (2)
- 00: Persons with incomplete or conflicting data (3)

## Categories created for the purpose of this study

Family type in this study was categorized as:

1. Cohabitation
2. Not cohabitation
3. Missing data

## Comments

According to Statistics Sweden, there may be difficulties in identifying cohabiting individuals who do not have children together. In this study, however, the requirements for this were equal for both groups.

## Data about the populations with baseline years

Between 2008 and 2015

# 5) Highest achieved Educational Level

## Source of data (which registry at Statistics Sweden)

LISA, which gathers data on the highest achieved educational level from the Register on Participation in Education at Statistics Sweden. Schools and education providers in the country continuously report data to this register. Information from individuals who have immigrated to Sweden is gathered for the register through questionnaires.

## Type of data delivered from Statistics Sweden and its description

Education is classified according to the Swedish Educational Terminology (SUN2000), which is the Swedish version of the international classification ISCED 97 (International Standard Classification of Education). SUN2000 consists of two modules, a level module and an alignment module, which describe the highest achieved educational level of an individual. The level module has a hierarchic 3-digit code, where the first digit expresses the level of education, the second its length in years and the third the type of education. The definitions of the first two digits in the level module are listed below. The figure in parentheses describes how the data was categorized in this study.

- 00: preschool education (1)
- 10: primary education <9 years (1)
- 20: primary education 9 years (1)
- 31: secondary school <2 years (2)
- 32: secondary school 2 years (2)
- 33: secondary school 3 years (2)
- 41: postsecondary education <2 years (2)
- 52: postsecondary education 2 years (2)
- 53: postsecondary education 3 years (3)
- 54: postsecondary education 4 years (3)
- 55: postsecondary education ≥5 years (3)
- 60: other/unspecified research education (3)
- 62: licentiate education (3)
- 64: PhD education (3)

## Categories created for the purpose of this study

In this study, the first two digits of the code in the level module (level and years of education) were used and categorized as follows:

1. Low (≤primary school [0–9 years])
2. Medium (secondary school up to postsecondary education <3 years [10–14 years])
3. High (postsecondary education ≥3 years [≥15 years])

## Comments

Because education could be considered a stable variable that did not change in the populations in this study, the highest achieved educational level at 2015 was used for the population with baseline 2016.

## Data about the populations with baseline years

Between 2008 and 2016

# 6) Employment

## Source of data (which registry at Statistics Sweden)

LISA, which collects data on employment from the register-based labor market statistics (Swedish acronym RAMS) at Statistics Sweden. RAMS uses different sources to determine whether an individual is employed. The main sources are control data from employers and information from tax assessment material (for self-employed). From this data a threshold value of income is used to determine whether an individual is to be defined as being employed. Specific benefits from the Swedish Social Insurance Agency (SSIA) such as sickness benefits, maternity allowance and parental benefits for child care, also form the basis for being assessed as employed.

## Type of data delivered from Statistics Sweden and its description

Data was delivered as two different variables: SyssStatJ for the population with baselines during 2008–2011 and SyssStat11 for the population with baselines during 2011–2015 (see details under Comments). Both variables were categorized into the following three groups. The figure in parentheses indicates how the data was categorized in this study.

- - - 1: Employed aged 16–74 years (aged 16–84 years for variable SyssStatJ) (1)
    - 5: Unemployed, with control data from employers or information from tax assessment material (for self-employed) (2)
    - 6: Unemployed, without control data from employers or information from tax assessment material (for self-employed) (2)

## Categories created for the purpose of this study

The two variables were merged together, SyssStatJ for the population with baselines between 2008 and 2010 and SyssStat11 for the population with baselines between 2011 and 2015, to create a new variable for employment which was categorized as:

1. Employed
2. Unemployed/retired (>65 years)

## Comments

In 2011, Statistics Sweden made some methodological changes in the definition of being employed. The upper limit for being classified as employed was changed from 84 to 74 years of age. The methodological changes also affected the self-employed over 65 years, to simplify categories so that they were still defined as employed after the age of general retirement in Sweden (>65 years) up to the age of 74. Because there is no translation key between these changed classifications, Statistics Sweden delivered both variables (SyssStatJ and SyssStat11) for the populations that had their baseline during 2011. When we compared these two variables, we identified that only a smaller number of the BOA population was affected (3.5%); therefore, we chose to merge the variables and analyze them as a single variable.

## Data about the populations with baseline years

Between 2008 and 2015

# 7) Occupation

## Source of data (which registry at Statistics Sweden)

LISA, which collects data on occupation from the Swedish Occupational Register at Statistics Sweden. To classify occupation, information is gathered from approximately 20 different sources, although the most important sources are statistics relating to salary structures in the public and private sectors, employer organizations and questionnaires to employers.

## Type of data delivered from Statistics Sweden and its description

Since 2014, Statistics Sweden uses the Standard Swedish Classification of Occupations, called Ssyk2012, to classifying occupations using a 4-digit code. This is the Swedish version of the International Standard Classification of Occupations from 2008 (ISCO-08), created by the International Labour Organization. An earlier version of the classification is called Ssyk96 (ISCO-88). For this reason, data was delivered from Statistics Sweden as two variables: Ssyk96 for the population with baselines between 2008 and 2013 and Ssyk2012 for the population with baselines between 2014 and 2015 (see details under Comments).

Both Ssyk96 and Ssyk2012 were delivered as 3-digit codes, where the first digit indicates the occupational group from 10 groups (in both versions) based on the type of work, its tasks and the skill required. The different occupational groups are described similarly in the two versions:

- **Ssyk96**
  - 0: Armed forces occupations
  - 1: Management
  - 2: Occupations requiring special theoretical skills
  - 3: Occupations requiring shorter university education
  - 4: Office and customer occupations
  - 5: Service, care and shop sales occupations
  - 6: Agricultural, gardening, forestry and fishery occupations
  - 7: Construction and manufacturing trades
  - 8: Process and machine operator occupations, transport occupations, etc.
  - 9: Work without the need for special vocational training
- **Ssyk2012**
  - 0: Armed forces occupations
  - 1: Managers
  - 2: Occupations requiring an advanced level of higher education
  - 3: Occupations requiring higher education qualifications or equivalent
  - 4: Administration and customer service clerks
  - 5: Service, care and shop sales occupations
  - 6: Agricultural, horticultural, forestry and fishery occupations
  - 7: Building and manufacturing occupations
  - 8: Mechanical manufacturing and transport occupations, etc.
  - 9: Basic occupations

## Categories created for the purpose of this study

The two variables (Ssyk96) for the population with baseline between 2008 and 2013 and Ssyk2012 for the population with baseline between 2014 and 2015) were merged to create a new variable of occupation that was categorized as:

- white-collar occupations (nonmanual labor) (areas 0–4)
- blue-collar occupations (manual labor) (areas 5–9)

## Comments

Analysis of the categorization of specific occupations in the two versions based on the 4-digit code showed that some occupations had been changed from white-collar to blue-collar and vice versa between versions. Although a translation key between the two versions exists, Statistics Sweden regards this as not completely reliable. Therefore, we made a choice to define a particular type of occupation as white- or blue-collar work depending on how the specific occupation was defined at the time of each individual’s baseline.

In some of the analyses in this study, the populations were grouped by age into ≤65 years and >65, because the general age of retirement in Sweden is 65 years. Because Statistics Sweden reports that data concerning the occupation of individuals >64 years is less reliable, we chose to analyze the SES indicator occupation only among participants who were classified as employed and were ≤65 years at baseline.

## Data about the populations with baseline years

Between 2008 and 2015

# 8) DISPOSIBLE Income

## Source of data (which registry at Statistics Sweden)

LISA, which collects data on an individual’s disposable income from the Income and Taxation Register at Statistics Sweden. Disposable income is defined as that part of an individual’s income that can be used for consumption or saving. It includes income from employments, social welfare, pension (public and private), sickness benefits and several other benefits that are handled by SSIA. It also includes income from business activities and income from capital minus taxes and deductions.

## Type of data delivered from Statistics Sweden and its description

Statistics Sweden delivered data on disposable income per consumption unit in the household for the individual’s baseline year in the study and for 3 years prior to that year. Disposable income per consumption unit in the household is calculated by Statistics Sweden by dividing the sum of all disposable income of the family members by the total consumption weight of the family (see the definitions of various family types above). The consumption weight was developed by The National Board of Health and Welfare, which weighted 1 adult in the household as 1.16, two adults as 1.92 and children as 0.56–0.76, depending on their age.

## Categories created for the purpose of this study

The household disposable income was categorized in quartiles, which were calculated from the mean income over the period (baseline year and 3 years prior) for the reference population. These quartiles were created to allow the comparison of the different cohorts (e.g., those with knee or hip osteoarthritis) with their matched reference individuals. When reporting the results in this study, disposable income is expressed in Euro (€). During January to September 2019, the average value was: 1.00 Swedish kronor (SEK) = 0.095 € [3].

- Lowest quartile: ≤12,262 €
- Second quartile: 12,263-16,654 €
- Third quartile: 16,655-22,962 €
- Highest quartile: ≥22,963 €

The disposable income of the household was also reported as median (IQR), because the populations were not normally distributed.

## Comments

The BOA population and the reference population had small but similar differences in their change in disposable income during the years before baseline; thus, only results from the baseline year are presented in this study.

## Data about the populations with baseline years

Between 2008 and 2015

# 9) Sick leave

## Source of data (which registry at Statistics Sweden)

LISA collects data about sick leave from SSIA, which administers, pays and records sick leave. In Sweden, there is no compensation for the first day in a period of sick leave. Days 2–14 of sick leave are paid by the employer and are not registered by SSIA. From day 8 of a sick leave period, a sick leave certificate issued by a medical doctor is required to receive compensation. In Sweden you can be entitled to either part-day or full-day sick leave.

## Type of data delivered from Statistics Sweden and its description

Statistics Sweden delivered data from an individual’s baseline year in the study and 3 years prior to that year, as days and numbers of periods of sick leave (only from day 15, when SSIA starts to compensate the person on sick leave).

Data for gross and net days of sick leave and periods of sick leave was delivered for each reported year.

- Gross day: one sick leave day is counted as one calendar day regardless of whether it is part time or full time
- Net day: e.g., sick leave comprising 50% of a day is calculated as 0.5 days of sick leave.

## Categories created for the purpose of this study

In this study, only gross days of sick leave were analyzed and reported for the baseline year plus the 3 years prior to baseline.

## Comments

It was not possible to follow sick leave periods that lasted more than one calendar year because a date was not recorded for all the included years.

## Data about the populations with baseline years

Between 2008 and 2015

# References

1. Ludvigsson JF, Almqvist C, Bonamy AK, Ljung R, Michaelsson K, Neovius M, et al. Registers of the Swedish total population and their use in medical research. European journal of epidemiology. 2016;31(2):125-36; doi: 10.1007/s10654-016-0117-y.

2. The Swedish Association of Local Authorities and Regions (SALAR). Classification of Swedish municipalities 2017. https://skl.se/download/18.6b78741215a632d39cbcc85/1487772640274/Classification%20of%20Swedish%20Municipalities%202017.pdf. 2019-10-24.

3. Swedish Riksbank. Monthly average exchange rates. https://www.riksbank.se/sv/statistik/sok-rantor--valutakurser/manadsgenomsnitt-valutakurser. Accessed 24 October2019.
